# Equine fatalities in equestrian eventing

**DOI:** 10.1111/evj.14529

**Published:** 2025-05-15

**Authors:** Heather A. Cameron‐Whytock, Denzil O'Brien, Victoria Lewis, Tim Parkin, Euan D. Bennet

**Affiliations:** ^1^ School of Veterinary Medicine University of Central Lancashire Preston Lancashire UK; ^2^ PO Box 1167 Balhannah South Australia Australia; ^3^ Equestrian Performance Research and Knowledge Exchange Arena Hartpury University Hartpury UK; ^4^ Bristol Veterinary School University of Bristol Bristol UK; ^5^ School of Veterinary Medicine University of Glasgow Glasgow UK

**Keywords:** cross‐country, eventing, fatality, horse, safety, sudden death

## Abstract

**Background:**

To date, industry and research outputs that have aimed to improve safety in equestrian eventing have tended to focus on identifying risk factors for horse falls during cross‐country, which have been identified as the greatest risk of injury and fatality for riders. There is an absence of research that investigates fatalities of horses within the sport of eventing.

**Objectives:**

To use a combination of media reports and eventing federation databases to describe and document equine fatalities in equestrian eventing, including their context, location and a basic pathology.

**Study Design:**

Descriptive study.

**Methods:**

A study describing equine fatalities that occurred between 1998 and 2023. Study data were taken from a combination of media reports (to identify occurrences of equine fatalities) and federation databases (to confirm a fatality did indeed occur and validate data).

**Results:**

One hundred and ten equine fatality records were included. Median horse age was 12 years. 62.7% of equine fatalities were not associated with a report of a horse fall during the cross‐country phase. Of all fatalities, 36.4% involved a musculoskeletal (MSK) injury, 36.4% were considered sudden death and 27.3% did not report the pathology (unknown). A total of 47.5% of MSK‐related fatalities and 90% of sudden death fatalities occurred during cross‐country but were unrelated to horse falls at cross‐country fences.

**Main Limitations:**

The data presented within this study do not provide a complete picture of global equine eventing fatalities during the study period. Details around the context/pathology of fatalities are also limited because of the sources they are derived from.

**Conclusions:**

This study demonstrates that equine fatalities in eventing occur in contexts other than horse falls, including as a result of MSK injury and sudden death. Future research and risk management work in eventing should include work that investigates sudden death and MSK injury in eventing horses and their causative/associative factors.

## 1 INTRODUCTION

Eventing is one of three Olympic equestrian disciplines and includes three distinct phases: dressage, cross‐country and jumping. To date, industry and research outputs that have aimed to improve safety in the sport of equestrian eventing have tended to focus on identifying risk factors for horse falls during the cross‐country phase of the sport, which have been identified as the greatest risk of injury and fatality for riders.^1^, ^2^, ^3^, ^4^, ^5^, ^6^, ^7^, ^8^ Horses within the sport are also at risk of injury and fatality; however, the risks are currently not well understood because of challenges in data capture.

In Thoroughbred racing jurisdictions with strict mandatory reporting and recording requirements of all equine fatalities, many academic studies have investigated fatalities associated with racing. Two types of fatality have been investigated extensively in the literature: musculoskeletal (MSK) injuries and sudden death. Fatalities due to MSK have an incidence of around 1.0–2.0 fatalities per 1000 race starts previously reported, depending on location.^9^, ^10^, ^11^, ^12^, ^13^ Sudden death is a comparatively rare outcome, occurring in around 1 in 10,000 race starts.^14^, ^15^, ^16^, ^17^, ^18^

Annual reports which outline the incidence of horse falls in eventing competition (including the level of injury sustained by riders in such events) are publicly available, for example from the world governing body for equestrian sport, the Fédération Equestre Internationale (FEI). Notably, statistics on horse injury during eventing competition are absent within these reports, and perhaps because of this, research that specifically focuses on the risk of injury and fatality to eventing horses is also scarce. For the first time since the publication of FEI eventing summary statistics, the FEI included data on equine fatalities related to cross‐country fences in their 2024 issue, with the number of equine fatalities per year from 2023, dating back to 2012, including the level of competition at which they occurred. No other details are provided however, and since only fatalities related to a cross‐country fence are included in the report, the data are incomplete.^19^ Currently, the only publicly available source of information on all types of eventing‐related horse injury and fatality is via online media reports, which typically include greater detail regarding the location and context of the fall, and demographics of the horse. Due to the lack of available data on equine fatalities in eventing, it is reasonable to assume that risk factors for injuries and fatalities in eventing horses are not yet definitively understood.

Both national and international governing bodies of eventing have worked to improve safety within the sport, particularly through policy and rule changes designed to improve the welfare of the horse and the safety of both horses and riders. However, the extent to which these changes have been based on evidence from peer‐reviewed scientific literature is unclear. In 2022, the FEI created the Equine Ethics and Wellbeing Commission, with the aim of receiving independent advice and recommendations from the group relating to safeguarding horse welfare within the sport,^20^ which may prompt further advances in the sport.

The social licence to operate for equestrian sport continues to be under increasing scrutiny, with governing bodies under immense pressure to demonstrate that the welfare of the horse is a priority. It is therefore imperative that the mechanisms of injury and fatality for equine eventing participants are investigated, to set plans for future research and enable improvements to be made. The aim of the current study was therefore to provide an overview of equine fatalities in eventing, including their context, location and descriptive pathology. This work is the first of its kind in scientific literature, with no previous studies available that describe the epidemiology of eventing‐related equine fatalities. Work such as this has the capacity to highlight the need to improve the safety, wellbeing and welfare of eventing horses and positively impact the social licence of the sport.

## 2 MATERIALS AND METHODS

Study data were harvested from online media reports of equine fatalities in eventing. Inclusion criteria were set a priori as fatalities that occurred during or immediately after eventing competition (any level, affiliated). Fatalities from all available years were included.

The media outlet HorsetalkNZ have a dedicated webpage related to both horse and rider fatalities in eventing, which contains a list of links to media reports in which fatalities are described, this webpage was a key source for identifying relevant media reports.^21^ Exploratory searches were also performed using online search engine Google™, to identify additional media reports of equine fatalities. Where reports of a fatality were covered in more than one media outlet, the data were compared, combined and extracted from all sources. Where media reports contained contradictory information regarding the same variable, relating to the same fatality, these data points were labelled as ‘unknown’ in the data set. Only media reports that could be verified using publicly availably National Federation or FEI databases were included, to ensure validity of included data. Horse name, level of competition and competition location/name were key identifying factors used as verification. Any media report that could not be verified (reconciled) with a National Federation/FEI database was excluded.

When the reported circumstances were that the horse ‘collapsed’, either with or without a clear pathological cause of death, the fatality was classified as a ‘sudden death’. This is consistent with the definition of sudden death used in the literature for Thoroughbred racing.^14^ Even with a full post‐mortem examination, it can be difficult to identify a specific cause of sudden death.^17^ Hence for this study, for any fatality which involved circumstances where the horse ‘collapsed’ but with unknown cause of death, the pathology was described as ‘sudden death’.

### 2.1 Descriptive analysis

Descriptive information on event level, circumstances of incident, pathology, type of fall, location of incident, horse age, horse sex and year of incident was derived from the harvested data, with percentage (of total fatality cohort) provided. Data are presented in aggregate form to mitigate identification of individuals. All reported proportions include a 95% confidence interval calculated using the Wilson score interval.^22^

## 3 RESULTS

The initial study cohort identified included 176 reported equine fatalities which occurred between 1998 and 2023. The following inclusion criteria were applied: first, any fatality that could not be validated by confirmation of competition records on FEI or national federation websites was excluded. In total, 60 reported fatalities were not verifiable with publicly available competition records; these were excluded from the final study cohort. Second, any fatality that was reported to have occurred outside of competition—that is, that was described as occurring either in training, elsewhere, or otherwise unknown—was removed: as a result, six further fatalities were excluded from the final study cohort.

The final study cohort consisted of 110 equine fatality records. The median horse age was 12 years (interquartile range 10–15, minimum 5, maximum 24). Of the recorded fatalities, 77.3% (95% confidence interval [CI] 68.6%–84.1%, *n* = 85) occurred at FEI (international level) events; the remaining 22.7% (CI 15.9%–31.4%, *n* = 25) occurred at national level competitions. Table 1 shows descriptive statistics of fatalities by several factors which could be identified from most media reports. The year 2018 had the highest number of fatalities with 10.9% (CI 6.3%–18.1%, *n* = 12). Of the recorded fatalities, 69.1% (CI 59.9%–77.0%, *n* = 76) involved a fence; however, only 33.6% (CI 25.5%–42.9%, *n* = 37) involved a horse fall at a fence. Sudden death was recorded as the cause of death in 30.9% (CI 23.0%–40.1%, *n* = 34) of cases. The highest prevalence was recorded in the USA with 39.1% (CI 30.5%–48.4%, *n* = 43).

**TABLE 1:** Total number of fatalities recorded by several factors.

Factor	Number of recorded fatalities	Percentage of all recorded fatalities	95% confidence interval
All horses	110	100.0%	
Year of incident			
1998	1	0.9%	0.2%–5.0%
2004	1	0.9%	0.2%–5.0%
2007	6	5.5%	2.5%–11.4%
2008	9	8.2%	4.4%–14.8%
2009	6	5.5%	2.5%–11.4%
2010	7	6.4%	3.1%–12.6%
2011	3	2.7%	0.9%–7.7%
2012	8	7.3%	3.7%–13.7%
2013	6	5.5%	2.5%–11.4%
2014	7	6.4%	3.1%–12.6%
2015	6	5.5%	2.5%–11.4%
2016	9	8.2%	4.4%–14.8%
2017	8	7.3%	3.7%–13.7%
2018	12	10.9%	6.3%–18.1%
2019	2	1.8%	0.5%–6.4%
2020	3	2.7%	0.9%–7.7%
2021	4	3.6%	1.4%–9.0%
2022	7	6.4%	3.1%–12.6%
2023	5	4.5%	2.0%–10.2%
Event level			
1*	7	6.4%	3.1%–12.6%
2*	10	9.1%	5.0%–15.9%
3*	36	32.7%	24.7%–42.0%
4*	28	25.5%	18.2%–34.3%
5*	3	2.7%	0.9%–7.7%
Olympics/World Equestrian Games	2	1.8%	0.5%–6.4%
Total international level	85	77.3%	68.6%–84.1%
Novice	9	8.2%	4.4%–14.8%
Intermediate	9	8.2%	4.4%–14.8%
Advanced	6	5.5%	2.5%–11.4%
Total national level	25	22.7%	15.9%–31.4%
Fence involved			
No	34	30.9%	23.0%–40.1%
Yes	76	69.1%	59.9%–77.0%
Horse fall involved			
No	69	62.7%	53.4%–71.2%
Yes	41	37.3%	28.8%–46.6%
Rotational horse fall involved			
No	75	68.2%	59.0%–76.2%
Yes	13	11.8%	7.0%–19.2%
Unknown	22	20.0%	13.6%–28.4%
Sudden death			
No	76	69.1%	59.9%–77.0%
Yes	34	30.9%	23.0%–40.1%
Location of incident			
On XC course	89	80.9%	72.6%–87.2%
After XC	13	11.8%	7.0%–19.2%
On SJ course	1	0.9%	0.2%–5.0%
Unknown	7	6.4%	3.1%–12.6%
Country where incident occurred			
USA	43	39.1%	30.5%–48.4%
Great Britain	31	28.2%	20.6%–37.2%
France	6	5.5%	2.5%–11.4%
Germany	6	5.5%	2.5%–11.4%
Ireland	6	5.5%	2.5%–11.4%
Australia	5	4.5%	2.0%–10.2%
Canada	3	2.7%	0.9%–7.7%
Greece	3	2.7%	0.9%–7.7%
New Zealand	3	2.7%	0.9%–7.7%
Poland	3	2.7%	0.9%–7.7%
Japan	1	0.9%	0.2%–5.0%

Table 2 shows descriptive statistics for the reported pathology of all recorded fatalities, with an equal number of sudden death and MSK at 36.4% (CI 28.0%–45.7%, *n* = 40) and 27.3% (CI 19.8%–36.3%, *n* = 30) pathology unknown. Table 3 shows the pathology of equine fatalities by the presence or absence of a horse fall at a fence reported as part of the incident, with the majority (52.5%, CI 37.5%–67.1%, *n* = 21) of MSK‐related fatalities involving a horse fall at a fence. On the contrary, most (90%, CI 77%–96%, *n* = 36) sudden death‐related fatalities did not involve a horse fall at a fence.

**TABLE 2:** Table of pathology as described in media reported eventing horse fatalities.

Pathology	Number of recorded fatalities	Percentage of all recorded fatalities	95% confidence interval
Musculoskeletal injury	40	36.4%	28.0%–45.7%
Sudden death	40	36.4%	28.0%–45.7%
Unknown	30	27.3%	19.8%–36.3%

**TABLE 3:** Table of pathology by the presence or absence of a horse fall involved in the reported incident.

Pathology	No horse fall involved (%, 95% confidence interval [CI])	Horse fall involved (%, 95% confidence interval [CI])
Musculoskeletal injury	19 (47.5%, CI 32.9%–62.5%)	21 (52.5%, CI 37.5%–67.1%)
Sudden death	36 (90%, CI 77.0%–96.0%)	4 (10%, CI 4.0%–23.0%)
Unknown	14 (46.7%, CI 30.2%–63.9%)	16 (53.3%, CI 36.1%–69.8%)

## 4 DISCUSSION

The aim of the study was to provide an overview of equine fatalities in eventing, including their context, location and pathology. Scientific literature, governing body reports and audits have previously focused on the reduction of horse falls to improve safety in the sport, which are certainly the predominant factors for rider injury and fatality.^1^, ^2^, ^3^, ^4^, ^5^, ^6^, ^8^ The current study has identified that while risk management strategies have indeed focused on improving equine safety and welfare, such strategies should also begin to consider non‐fall‐related injuries and fatalities as a point of interest for reducing equine injury and fatality.

The number of fatalities recorded per year appears to be lower between 1998 and 2006. However, this may be due to either a lack of media reporting during those years, or because some media reports that were available have now been archived. There were a lower number of reported fatalities between 2019 and 2022 in comparison with the preceding 12 years, which could indicate that changes in policy in relation to risk management have been successful in reducing the risk of equine fatalities. Notably, however, an upward trend is apparent since 2020, when competition was affected by the COVID‐19 pandemic; therefore, the forthcoming years will need to be carefully monitored to confirm whether consistent improvements are apparent. It is also important to note that no denominator (i.e., number of starts) data was available for this study, so increases in the number of fatalities may also, in part, be related to more horses competing.

The majority of equine fatalities reported in the current study occur at higher levels of competition such as 3* and 4* international competition, indicating there are more equine fatalities at the higher levels of competition. Increased risk of horse falls at higher levels of eventing competition has been previously reported in empirical studies; therefore, it could be assumed that this finding is comparable to previous research. However, it is important to note that those studies did not consider equine injuries and sudden death in relation to horse falls specifically.^1^, ^2^, ^3^, ^4^, ^5^, ^6^, ^8^ Conversely, considering the source of the data in the current study (media articles), this finding may be influenced by bias in media reporting, where the higher levels of competition are more likely to have equine fatalities reported in the media due to the high‐profile nature of the competition, the riders and the horses involved. It is vital to note that this study likely does not provide a complete picture of equine fatalities in eventing as such.

When information was available on whether a fence was involved in a fatality, it appears that the majority of fatalities did involve an incident at a fence. This suggests that, as previously believed, fences are a key factor to consider in risk management strategies to reduce the risk of injury and fatality to both the horse and the rider.^1^, ^2^, ^3^, ^4^, ^5^, ^6^, ^7^, ^8^ Notably, the majority of literature that has investigated fence‐related risk has focused on risk factors for horse falls.^1^, ^2^, ^3^, ^4^, ^5^, ^6^, ^8^ It is strongly recommended that future work begin to investigate fence‐related risk factors for equine injury, independent of horse falls, which may present unique, previously unidentified characteristics of fence and course design that could reduce the risk of injury and fatality to horses. For example, fences of certain designs may require horses to move (i.e., approach, or jump) in sub‐optimal locomotory patterns, thus increasing the risk of MSK injury. Alternatively, certain fence designs or materials may increase the risk of soft tissue/MSK injuries because of their inherent structure or surface characteristics. Additionally, the characteristics of cross‐country surfaces (where fences are sited) may impact equine injury risk, warranting further investigation.^23^

This study has identified that sudden death—as defined in previous studies of Thoroughbred racing—is a predominant cause of equine fatalities in eventing. Without making direct comparisons between disciplines, in Thoroughbred racing in North America there were 7220 horse fatalities (all causes) recorded between 2009 and 2022, and of those only 7.4% (*n* = 536) had the cause recorded as sudden death.^14^ With the information available for the present study, at least 36.4% (CI 28.0%–45.7%, *n* = 40, with the pathology of another 30 fatalities unknown) of recorded fatalities in eventing were attributed to sudden death, by the same definition used in.^14^ Despite this, there is a lack of discourse around sudden death in eventing in both industry and scientific literature. The present study found at least 36.4% (CI 28.0%–45.7%, *n* = 40, with 30 unknown) of recorded fatalities in eventing had pathology consistent with the definition of ‘musculoskeletal injury’ (MSK) used in Thoroughbred racing. Excluding the 30 fatalities with unknown pathology, the proportions of fatality by type described in the present study were 50% MSK, 50% sudden death (95% confidence intervals 39.3%–60.7% for both types). Given the lack of previous investigation of equine fatalities in eventing, it is impossible to speculate whether the expected proportion of fatalities should be the same as or different from the 92.6% MSK, 7.4% sudden death reported in racing by.^14^ However, this finding would suggest that, as a proportion of all fatalities, sudden death is more prominent in eventing than in racing and that urgent further investigation is needed into the potential mechanisms for sudden death in eventing, in order to establish the true prevalence.

The majority of incidents occurred on the cross‐country course, with the second most common location being after the cross‐country phase was completed. This finding indicates that the cross‐country aspect of eventing is indeed the most dangerous for horses in the sport. Risk management strategies should therefore continue to focus on the cross‐country phase of competition but should perhaps broaden the focus from horse falls to more general equine health. Human sports have implemented cardiovascular pre‐participation screening to prevent sudden deaths of athletes, which has been successful.^24^ The potential efficacy of protocols such as this should be investigated for horses participating in equine sport. Unofficial reports indicate that screening protocols for MSK injuries have been implemented in racing,^25^ suggesting that equestrian sport could move towards preventative cardiovascular and MSK screening in future, should the efficacy of such tools be proven.

The UK governing body for eventing sport (British Eventing) has since 2010 published an annual safety report on their website. The most recently available report includes information on the proportion of riders seriously or fatally injured during the 2021 season. However, similar information is not provided for horses.^26^ Similarly, the FEI publishes an annual statistics report, the most recent including information on the number of riders seriously or fatally injured, but no information on horse injury or fatality.^19^ Notably, the FEI report states that an improved follow‐up procedure was implemented in 2010 in order to have more reliable statistics, confirmed by the veterinary delegate, on the precise injuries sustained by horses.^19^ However, this information is not included in any publicly available reports to date.

While the reasons for the lack of information on equine injury and fatality provided by governing bodies are unknown, a number of reasonable assumptions can be made. For example, if an equine fatality occurs during one of the competition phases, such as the cross‐country, it is expected that the governing body can easily record such a case and the associated circumstances. If, however, a horse suffers an injury during or immediately after the competition, it may be transported home by the rider to receive veterinary treatment with the competitor's registered vet, and in these instances, it may be more challenging for the governing body to record the incident and the outcome as such (e.g., whether the horse successfully recovered following veterinary treatment, or was euthanised as a result of injuries). It is vital that the sport moves towards accurate reporting and sharing of equine injury and fatality data, to demonstrate transparency, improve public trust and evidence commitment to equine welfare, all of which are imperative to maintain the sport's social licence to operate.

Other challenges for the provision of accurate and representative data collection on equine injuries and fatalities in the sport may include the structure of the sport nationally and internationally, which sees National Federations gather data independently from the data gathering conducted by the world governing body (FEI), which only gathers data from international competitions. A collective approach where standards are set and governed by the FEI and disseminated to national federations would no doubt improve the quality and veracity of data collected on equine injury and fatality in the sport, which would in turn speed up any data analysis and potential policy/procedure changes that could occur.

Other horse sports have demonstrated how accurate data gathering on equine injury and fatality can be used to drive risk management strategy. A number of horse racing governing bodies have worked to improve their data gathering processes, which in turn have led to successful research projects to identify risk factors for equine injuries and fatalities. Following close engagement with research since 2009, Thoroughbred racing in North America has seen a 38% reduction in the incidence of equine fatalities (by all causes) as of 2022.^27^ Furthermore, the FEI has implemented evidence‐based rules for the sport of endurance.^28^, ^29^ Eventing governing bodies should consider adopting a comparable approach in order to achieve similar improvements to horse safety and welfare within eventing.

Social Licence to Operate is currently a primary topic of discussion regarding horse sport. Several areas of horse sport have already demonstrated a rounded approach to policy development, such as endurance and horse racing,^29^, ^30^, ^31^ as noted above. As evidence becomes available from science and research, equestrian sports must adapt and respond to improve. It is critical that scientific studies are commissioned to identify risk factors for all equine fatalities in eventing and that the information from such studies is embedded within future policy and regulatory change. Equestrian sports have the responsibility of mitigating risk and improving safety and welfare, using evidence as it becomes available.

## 5 CONCLUSION

Recent risk management in equestrian eventing has focused on reducing the risk of horse falls at fences, with the aim of mitigating the risk of injury and fatality to horses and riders within the sport. This focus on risk management within the sport is warranted but has perhaps overlooked important elements of fatality risk to horses specifically. This study demonstrates that equine fatalities in eventing occur in other contexts including those due to MSK injury and sudden death. Future research and risk management work in eventing should therefore look to include work that investigates sudden death and MSK injury in eventing horses and their causative/associative factors, which may identify beneficial policy/rule changes that could be implemented by sports governing bodies, stakeholders, riders or veterinarians to reduce those risks.

## FUNDING INFORMATION

Not applicable.

## CONFLICT OF INTEREST STATEMENT

The authors declare no conflicts of interest.

## AUTHOR CONTRIBUTIONS

**Heather A. Cameron‐Whytock:** Conceptualization; investigation; writing – original draft; writing – review and editing; validation; methtodology; data curation. **Denzil O'Brien:** Conceptualization; investigation; methodology; writing – review and editing; data curation. **Victoria Lewis:** Investigation; writing – review and editing; methodology. **Tim Parkin:** Investigation; writing – review and editing; methodology. **Euan D. Bennet:** Conceptualization; investigation; writing – original draft; writing – review and editing; data curation; methodology; validation.

## DATA INTEGRITY STATEMENT

Heather A. Cameron‐Whytock and Euan D. Bennet had full access to all the data in the study and take full responsibility for the integrity of the data and the accuracy of the data analysis.

## ETHICAL ANIMAL RESEARCH

Research ethics committee oversight not currently required by this journal: data sources are in the public domain.

## INFORMED CONSENT

Not applicable.

## PEER REVIEW

The peer review history for this article is available at https://www.webofscience.com/api/gateway/wos/peer‐review/10.1111/evj.14529.

## Data Availability

The data that support the findings of this study are available upon reasonable request from the corresponding author. Open data sharing exemption granted by the editor.
